# A qualitative exploration of daily path and daily routine among people in Ukraine who inject drugs to understand associated harms

**DOI:** 10.1186/s13011-022-00465-3

**Published:** 2022-05-07

**Authors:** Jill Owczarzak, Jessie Chien, Karin Tobin, Alyona Mazhnaya, Olena Chernova, Tetiana Kiriazova

**Affiliations:** 1 Johns Hopkins Bloomberg School of Public Health, 624 N. Broadway Ave, Room 739, Baltimore, MD 21205 USA; 2 Department of Community Health Sciences, UCLA Fielding School of Public Health, 650 Charles E. Young Drive South, 36-071 CHS, Box 951772, Los Angeles, CA 90095-1772 USA; 3 Johns Hopkins Bloomberg School of Public Health, 2213 McElderry Street, 2nd Floor, Baltimore, MD 21205 USA; 4 School of Public Health, National University of Kyiv-Mohyla Academy, 10 Voloska Street, Building 6, 104-109, Kyiv, 02000 Ukraine; 5 Ukrainian Institute on Public Health Policy, 5 Biloruska Street, Kyiv, 04050 Ukraine

**Keywords:** Drug use, Daily routine, Ukraine, Harm reduction

## Abstract

**Background:**

Patterns of movement, heterogeneity of context, and individual space-time patterns affect health, and individuals’ movement throughout the landscape is shaped by addiction, meeting basic needs, and maintaining relationships. Place and social context enable or constrain behavior and individuals use social networks and daily routines to accomplish individual goals and access resources.

**Methods:**

This article explores drug use as part of daily routines and daily paths among people who inject drugs in Dnipro City, Ukraine. Between March and August 2018, we interviewed 30 people who inject drugs living in Dnipro City, Ukraine. Study participants completed a single interview that lasted between 1 and 2 hours. During the interview, participants described their daily routine and daily path using a printed map of Dnipro as a prompt. Participants were asked to draw important sites; give time estimates of arrival and departure; and annotate on the map the points, paths, and areas most prominent or important to them. Participants also described to what extent their daily routines were planned or spontaneous, how much their daily path varied over time, and how drug use shaped their daily routine.

**Results:**

We identified 3 major types of daily routine: unpredictable, predictable, and somewhat predictable. Participants with unpredictable daily routines had unreliable sources of income, inconsistent drug suppliers and drug use site, and dynamic groups of people with whom they socialized and used drugs. Participants with predictable daily routines had reliable sources of income, a regular drug dealer or stash source, and a stable group of friends or acquaintances with whom they bought and/or used drugs. Participants with somewhat predictable daily routines had some stable aspects of their daily lives, such as a steady source of income or a small group of friends with whom they used drugs, but also experienced circumstances that undermined their ability to have a routinized daily life, such as changing drug use sites or inconsistent income sources.

**Conclusions:**

Greater attention needs to be paid to the daily routines of people who use drugs to develop and tailor interventions that address the place-based and social contexts that contribute to drug-use related risks.

## Introduction

Ukraine continues to have one of the highest HIV infection rates in Europe, with most HIV infections among people who inject drugs (PWID) and their sex partners [1–4]. Rates of HIV infection among PWID remain persistently elevated in Ukraine, despite recent efforts to expand access to opioid agonist therapy (OAT) for people who use opioids, increase antiretroviral therapy (ART) coverage for PWID living with HIV, and scale-up syringe exchange programs—harm reduction strategies with demonstrated effectiveness at reducing HIV infection rates [5–7]. Social networks play an important role in HIV transmission and prevention [8, 9], including in Ukraine and Eastern Europe [10–15]. Social networks are embedded in place and are dynamic: their structure, function, and norms vary by place and activity [16–18]. Over the course of a day, PWID social interactions and activities occur at defined place-types (home, work, school, drop-in centers, gathering areas such as playgrounds or parks) along an individual’s pathway with social network members who encompass multiple, sometimes overlapping roles, such as sex or drug partners, friends, family, neighbors, and co-workers. Daily routines, or the activities individuals regularly engage, and daily paths, or the temporal and special contexts in which these activities occur [19], are mutually constitutive of access to health-producing and harm-exacerbating resources and environments.

An activity space approach, which explores the local areas in which individuals habitually move during the course of daily activities, posits that understanding where PWID sleep, work, relax, socialize and use drugs can deepen our understanding of where risky behaviors such as syringe sharing are more likely to occur and where to locate to harm reduction services such as syringe exchange programs [20]. Studies on daily routines, daily path, and activity space demonstrate that place and social context enable or constrain behavior, and reveal that individuals use members of their social networks and daily routines to access housing, employment, support, and other resources, and accomplish individual goals [21–24]. Whether daily routines are rigid, flexible, or chaotic may create barriers or opportunities to access services or exposure to risk exacerbating environments or situations [19, 25, 26]. The convergence of people, norms, and place can also shape harms for PWID, as demonstrated by the links between public injection, overdose, and HIV infection [27–31].

Previous studies on the relationship between place, social networks, and drug use have focused on quantitative dimensions of place, such as the correlation between measures of activity space distance and risk behaviors such as syringe sharing [20, 32, 33] and connections between discreet measures of economic deprivation of geographic region and drug type [23]. Less attention has been paid to patterns of movement, heterogeneity of context, and “space-time patterns of every individual” [34] that affect health or how individuals’ movement throughout the landscape is shaped by addiction, meeting basic needs, and maintaining relationships [35]. In this article, we explore places of drug injection as part of daily paths and daily routines among PWID in Dnipro, Ukraine. We explore how PWID decide where and with whom to inject within the context of their daily routine, and consider the social, economic, and other factors that shaped whether people use drugs at home, away from home, in private places, or in public spaces. We argue that a deeper understanding of the places and people that PWID encounter and interact with can be pathway for new intervention opportunities and improving the delivery of well-established interventions.

## Methods

### Data collection

Between March and August 2018, we interviewed 30 PWID living in Dnipro City, Ukraine. Dnipro, with a population of about 1 million people, is Ukraine’s fourth largest city and a large industrial center in the southeastern region. Dnipro is the main commercial and industrial center of eastern Ukraine and serves as a strategic junction between Ukraine, Russia, and other European countries. Between 11,000 and 30,000 PWID live in Dnipro, with an estimated HIV and HCV prevalence among PWID being 39.7 and 63.8% respectively [6]. Participants were recruited through direct contact from agencies that provide services to PWID; to include the perspectives of individuals who are not clients of service agencies, we also engaged in participant referral and street-based recruitment (at needle exchange points and drug use sites). Participant demographic, drug use, and residential city district were reviewed periodically to ensure that participants representing diverse substance use, geographic, age, and gender characteristics were included in the study. Potential participants were informed about the goals of the study and interested participants were screened for eligibility (being 18 years or older, living in the study city, and injecting drugs within the last 30 days). Individuals who agreed to participate made an appointment to complete the interview at a time and location convenient for them, typically in a private room at a local nongovernment organization. Eligible participants provided oral informed consent. Participants received 500 hryvnia (approximately 10 USD) to complete the interview. This study was approved by the institutional review boards at Johns Hopkins Bloomberg School of Public Health and the Ukrainian Institute on Public Health Policy.

Study participants completed a single interview that lasted between 1 and 2 hours. Interviewers were Dnipro residents familiar with the city’s geography and drug use. During the interview, participants described their daily routine and daily path using a printed map of Dnipro as a prompt. Participants were asked to describe their “typical day” during the week, starting from the moment they woke up. Participants were asked about variations to their daily routine (e.g., weekends, holidays) in a separate part of the interview. Participants were asked to draw important sites; give time estimates of arrival and departure; and annotate on the map the points, paths, and areas most prominent or important to them. Participants did not provide exact addresses or points on the map, only approximate locations of sites. They also described where and with whom they lived; and a typical day, including where they worked or earned money (legally or illegally), places where they relaxed and had fun, where they received health care and other services, places they avoided and the reasons, and where they purchased, prepared, and used drugs. Participants also described to what extent their daily routines were planned or spontaneous and how much their daily path varied over time. Participants also answered a set of standardized questions about current family situation, educational attainment, financial status, employment status, and current health conditions (HIV, HCV, TB).

### Analysis

Interviews were transcribed verbatim, translated from Russian or Ukrainian to English, and uploaded to MAXQDA for coding and analysis. A detailed description of the initial coding and analysis process have been published elsewhere [36]. During axial coding, categories and themes were explored in relation to each other, and broader themes that combined and transcended existing codes were identified. In this step of the analysis, a broader category of “daily path and daily routine” was created that included segments from the codes income-generation, drug procurement, injection, starting the day, home & apartment, and drug injection places. For each participant, a narrative was written that summarized their responses within the category of “daily path and daily routine.” Participants’ responses and experiences in these intersecting domains were then summarized to understand daily path and daily routine, and further analysis explored the relationship between participant demographic characteristics, drug use practices, and daily routines. For the maps, analysis began by coding the places participants marked on the map according to key activity spaces that paralleled domains from the interview guide, including drug market (directly from a dealer or through stashes), places avoided, drug preparation, drug injection, income generation, health care utilization, home and apartment, home district, and leisure time. After coding, all maps were visually inspected to identify patterns in daily routines and daily paths along domains such as geographic spread of the area participants moved in throughout the day and the number of different places visited throughout the day. Final analysis compared participant maps to identify daily path/daily routine patterns and explored key themes within each daily routine typology.

## Results

We interviewed 22 men and 8 women; the mean participant age was 37 years. Only one-third of participants were employed in the formal economy either full or part-time; a similar number (9 participants) indicated that they had enough money to meet basic needs. Only one participant indicated that she engaged in sex work to generate income or acquire drugs. Just under half (*n* = 13) were HIV-positive. Only two participants were enrolled in an OAT program at the time of the interview.

Our analysis revealed that the extent to which participants traveled across the city and where and with whom they spent their time, including drug use, varied significantly. These variations were driven by drug market factors (drug availability, how they obtained drugs and from whom), domestic circumstances (living situation and caregiving responsibilities), and employment status and the need to generate income in the formal and informal economies. Based on these circumstances, we identified 3 major types of daily path/routine: unpredictable (*n* = 12), somewhat predictable (*n* = 9), and predictable (*n* = 9). Below, we use individual case examples and exemplar quotes from participants in each daily routine category to illustrate how injection drug use is embedded within a daily path. All names are pseudonyms.

### Unpredictable daily routines

Daily routines characterized as unpredictable included participants who often had unreliable sources of income, inconsistent drug suppliers and places to use drugs, and dynamic groups of people with whom they socialized and used drugs. One of the primary drivers of an unpredictable daily routine was income generation, which often coincided with uncertainties around drug procurement, drug use sites, and drug use partners. Many respondents with unpredictable daily routines worked in the informal economy, including stealing, collecting scrap metal, driving unlicensed taxis, and day labor (primarily construction). When working in the informal economy, respondents often encountered different people throughout the day and day-to-day. For example, if Myron, a 40-year-old man, did not find work as a day laborer at a construction site, he would search for scrap metal wherever he could, including new construction sites, abandoned buildings, and old dumps. Even though he had a steady acquaintance with whom he collected scrap metal, he often encountered other PWID who were also collecting scrap. Myron said that such people were not consistent, but that he would buy and use drugs with about ten different acquaintances. They would pool the scrap they collected, send one person to the recycling center to exchange it for cash, and then immediately buy and use drugs:

I met them, we bumped into each other at metal recycling points or somewhere else in the city. You can see each other from a distance, as they say. When you lose your supplier, you look for someone from these permanent ones. You see a drug addict, you get acquainted with him and have a dialogue, you win his trust and naturally you chip in and buy [drugs] with him together.

Similarly, Oleg, 42 years old, was unemployed and made money “in any possible way,” including stealing and reselling goods and or injecting other people (in exchange for drugs or money). He spent most of his time “on the street” and “on the move.” He explained how these chance encounters related to drug purchase and use:

I met [my friend] by accident. Well, he was going for stuff *(drugs)*, and I was too. I say: “Let’s sell something now. We’ll come to the neighborhood, we’ll steal from someone, sell these things and go [to buy drugs], and it’ll be my treat.

Unpredictable daily routines often fostered larger networks of people with whom to potentially buy and use drugs, a greater number of places to buy and use drugs, and many places—both public and private—to inject. In these situations, PWID potentially interacted with a variety of individuals throughout the day. Engagement in the informal economy often meant that who showed up to a job site could change and they became acquainted through their shared goal of obtaining drugs. They often injected with whomever was “around” in a drug purchase or use site out of convenience, even if they preferred to use alone or privately at home. The unpredictable daily routines could also relate to periods of intense drug use and staving off withdrawal that demanded greater income generation than some wage work would afford.

### Predictable daily routines

Daily routines characterized as predictable included participants who often had reliable sources of income, a regular drug dealer or stash source, and a stable group of friends or acquaintances with whom they bought and/or used drugs. They often had highly circumscribed daily paths and did the same activities with the same people daily. One such participant was Tetiana, a 42-year-old woman who lived in a one-bedroom apartment with her boyfriend, who knew about her drug use. Tetiana did not work because her criminal record prevented her from being hired. Instead, she got money from her boyfriend and government child subsidies for her 14-year-old daughter who lived nearby with a relative. She had a network of people she knew with whom she pooled resources to purchase drugs and bought her drugs from a dealer that she went to school with. Sometimes she would inject with the people she bought drugs with, but her health condition often made it difficult to use drugs around other people:

R: Everything comes down to money … We will help each other, so to speak … All the time lately. Because, you understand, even 100 hryvnias a day for a person who doesn’t work … I can give [the drugs] to that person, and then I’ll use it on my own, and he’ll use it on his own. Well, it depends. There are people who I will definitely not bring home. But if it is an old friend, I can bring him home or sit there for half an hour and talk about something, drink tea...

I: Does it happen that you use *(drugs)* outside your home?

R: No, it’s difficult now … To inject in bruises - it’s difficult. It’s cold now, veins hide … I don’t inject in the thick veins, which you can quickly inject in … Sometimes you can mess around for an hour …. It’s stressful, of course.

She characterized her life as a “drab existence.” After her morning injection, she spent the day cleaning the house and looking at things on the internet and social media.

Other participants who were not formally employed developed similar strategies to secure some sort of steady income and small group of drug use partners that contributed to stability and predictable daily routines. Viktor lived in an apartment with his mother and characterized his own daily routine as something that “absolutely doesn’t differ.” He did not work and had no personal income (e.g., from a pension or disability benefits). Instead, he facilitated drug purchases for other people, who then paid him in drugs or cash. He bought and injected drugs with the same group of friends each day, rotating whose responsibility it was to procure the drugs, and typically bought from the same dealer.

The owner of that *malyas* [a concentrated drug precursor] and myself go to my place. That’s a person I trust. I know that he won’t bring the police to my place the next day. I’ve known him a long time, we are acquaintances. That is not the first time, nor a second time, nor the last time, hopefully … Let’s put it that way, I have three or four people who can be at my side at such time. If a dude approaches me in the street tomorrow saying, “Hey, let’s go buy some drugs,” that will absolutely never happen. I won’t get in a car with some Joe Schmoe and go buy drugs.

Participants with predictable routines had steady sources of income, such as through pensions or partners, and bought their drugs from the same known source each day. In addition, participants with predictable routines often (but not always) tried to use drugs at home and limit their interactions with other people, including other PWID.

### Somewhat predictable daily routines

Daily routines characterized as somewhat predictable had participants with some stable aspects of their daily lives, such as a steady source of income or a small group of friends with whom they always used drugs, but also experienced circumstances that undermined their ability to have a routinized daily life, such as ever-changing drug use sites or inconsistent income sources. Sergiy, for example, was 36 years old and worked full-time in a plastics manufacturing company. He used drugs 3–4 times per day, including at home, at work, and in public places. His job created relative stability in Sergiy’s daily life and drug use. Three of Sergiy’s coworkers also used drugs, and they pitched in money to buy stashes together, either before work or during lunch breaks. They would also use drugs together in the locker room at work. While his job and coworkers brough stability to his life in terms of an income source and some steady drug use partners, Sergiy also had elements of instability and unpredictability in his daily routine, particularly around where he used drugs and who might be present when he injected. Sergiy explained that if he was experiencing withdrawal, he would use drugs in the apartment of a friend who lived near where he bought drugs, or in the restroom of a fast-food restaurant or other publicly accessible restroom on his way home if he “wants to inject very badly.” When he was in these situations, fears about getting “caught” by his supervisors, mother (with whom he lived), police, or other people hanging around caused Sergiy to inject quickly.

Sergiy’s job brought some predictability to his drug use, particularly because he had a steady source of income and some regular people with whom he injected. Other people with somewhat predictable daily routines experienced work-related instability, such as seasonality, but had other aspects of their daily routine, including drug use, that were more consistent. For example, Petro worked as a parking attendant but the actual day-to-day work could be highly unpredictable and unstable. As an unsanctioned activity, the specific lots where he parked cars changed every day. While Petro preferred to work in the parking lot of the city’s largest bazaar, he would work anywhere in the city if necessary. This unpredictability countered his highly predictable and stable drug use patterns: he mostly used drugs by himself, typically at home, but would occasionally use drugs in the entrance halls of apartment buildings, abandoned buildings, construction sites, or the riverfront.

Ivan’s daily routine similarly combined elements of stability and unpredictability. He generated income through stealing and pickpocketing (primarily cell phones—“there’s simply no money in wallets”) with a friend that he had known since high school and who also used drugs. Ivan characterized their relationship as “close” as they “trust each other.” Ivan served as the “accomplice” and looked out for passersby and cops. Ivan had decided that his drug use was incompatible with legal, fulltime employment because no job he would be able to find as a person who uses drugs would pay him enough to support his drug addiction. Pickpocketing provided Ivan with stability in that he spent most of his days with the same person. However, they traveled all over the city to different second-hand shops, clothing stores, and markets to both steal and sell the stolen items. Because they moved constantly through the day, their drug purchase and use sites changed constantly:

Say that we’ve successfully stolen money before that … and during these thefts, we have some amount of drugs on us. We find a place where this can be used so that people wouldn’t see, we shoot up and continue stealing … [It can be in] some wooded area, an abandoned building, something like that … We come in, it’s quick, just 2 minutes, shoot up and leave.

Ivan and his friend bought drugs at different locations scattered across the city, but the small circle of people who he used drugs with remained consistent: in addition to using throughout the day with his accomplice, in the evenings, Ivan typically went home and used drugs with his girlfriend and sometimes the same friend with whom he pickpocketed during the day.

## Discussion

Analysis of participants’ daily paths and daily routines revealed three primary patterns—unpredictable, predictable, and somewhat predictable—and how these different levels of predictability shaped drug use practices. These daily paths and daily routines were shaped by income-generating strategies; the drug market and drug availability; and relationships with dealers, other PWID, and family. Income generation strategies were among the primary determinants of whether participants experienced predictable or unpredictable daily routines. Participants who had no steady income source, whether full-time employment, a pension, a partner’s income, or financial support from family members, entered the informal economy to generate cash for both buying drugs and daily living expenses. Some participants who earned income in the informal economy moved throughout the city daily and encountered large numbers of different people each day. Happenstance regarding where scrap could be collected and sold, where drug stashes were picked up, and who was at a job site on a given day shaped who people bought and used drugs with. In contrast, other participants encountered few—if any—new people throughout their day, had a relatively steady source of drugs, and often used drugs alone or with the same small group of people.

Previous research has documented that unemployment among PWID is common and that without access to formal employment, PWID engage in informal or illegal income generation activities such as recycling, theft, and sex work [37, 38]. Research has also documented the (perceived) incompatibility between drug use (especially higher intensity) and formal employment. Lack of steady, predictable income is intertwined with potential drug-use related harms [39]. Ferguson (2015) found that engagement in illegal or informal income generation was associated with higher risk drug use among homeless young adults [40]. Cheng et al. (2016) found that 82% of street-involved youth who used drugs engaged in risky income generating strategies, which included sex for money, salvaging, panhandling, theft, and selling drugs, and that involvement in such risky strategies was associated with higher intensity drug use, experiencing violence, and interactions with police [41]. One proposed association between engagement in the informal economy, daily path/routine, and drug use associated harms is that PWID with less predictable routines may have more spontaneous and riskier drug use for which they are not prepared with clean drug use equipment (syringes, needles, cookers), and strong norms of not sharing used injection equipment may not be established among people who inject together occasionally or only once. Prior work has documented that existing policies in Ukraine, such as the requirement that PWID formally register with the governmental addiction clinic in order to receive free drug treatment such as medication assisted therapy, negatively impact PWID health and well-being [42–45]. In addition, for those PWID who *are* able to obtain work, employment may be in low-paid, unstable industries that may not pay enough to cover the costs of continued drug use. Interventions that address employment and economic opportunities to reduce poverty and increase socioeconomic stability among PWID could have potential health benefits, including HIV risk reduction [46].

Our analysis of PWID daily routines indicates that Dnipro lacked a specific “drug scene,” or specific geographical inner-city areas characterized by high concentrations of drug users [39]. While some participants in this study, particularly those involved in the informal economy, referred to places with high concentration of PWID, many described more diffuse drug purchase and consumption sites and buying and using drugs close to their homes or near income-generation sites. Whether their daily routines were predictable or unpredictable, most participants lived in the same neighborhood for dozens of years. The diffuse nature of drug use sites in Dnipro suggests that harm reduction services, including injection equipment vending machines, needle exchange sites, safe disposal and sharps containers, and OAT clinics [47], should be located throughout the city, including in residential neighborhoods, and with hours and access points that allow PWID to incorporate their use into their daily path and daily routine without extensive travel. Homes and residential neighborhoods served as sources of stability in otherwise unpredictable lives. Increasing accessibility of harm reduction services such as OAT is especially critical, as the OAT uptake remains suboptimal [48] and the number of OAT clinics remains low [49].

## Limitations

This study has several limitations. First, the Ukrainian drug use context is somewhat unique. The drug market has thus far been largely unaffected by the introduction of fentanyl and other synthetic opioids that has penetrated the US and other drug markets [50, 51]. The Ukrainian drug market may also differ from other settings in terms of the ways drugs are bought and procured, including purchases facilitated through banking apps and stashes and robust homemade drug production [52]. Second, all participants were currently housed and few of them experienced the chronic housing instability that characterizes the experiences of PWID about which much of the public injection literature describes [20, 47, 52–54]. Finally, in some instances, participants’ visual depiction of their daily path conflicted with the way they narratively described their day, for example characterizing their activities as taking them “all over the city” when their maps showed their activities as highly concentrated in a specific district. Recommendations for where to locate services for PWID should account for this limitation and consider the difficulty of crossing even a district within a large city on foot. Perceptions of distance and perceived burden of accessing services based on their location should be further explored. Other methodological approaches, such as an ethnographic component in which an ethnographer accompanies participants throughout their daily routines, could corroborate and contextualize participants’ recollection of events and produce a deeper understanding of the relationship between space, people, and drug use [55–57].In addition to these limitations, the extent to which these findings can be applied to other contexts, including PWID who live in more rural areas or smaller towns, should be consider. However, the theoretical and methodological consideration of understanding how space, relationships, and drug use intersect can be applied to diverse geographic settings.

## Conclusion

The framework of daily path and daily routine as applied to drug use practices indicates that not all PWID face the same vulnerabilities and that these different potential harms are shaped by economic, place-based, and social factors. This study indicates that greater attention needs to be paid to the daily routines and lived experiences of PWID to develop and tailor interventions that address the place-based and social contexts that contribute to drug use-related risks. The link between income generating strategies, daily path, and drug use suggests that PWID in Ukraine are a “structurally vulnerable population:” they occupy a marginal social position within the labor market, shaped by discrimination and stereotypes about PWID and policies that exclude them from the labor market, that in turn shape opportunities and produce disparities in health and social outcomes over time [37]. Understanding daily paths and daily routines of PWID can inform drug policy and programs, including appropriate location of harm reduction services, to increase their accessibility and effectiveness.

## Data Availability

The datasets generated during and/or analyzed during the current study are not publicly available due to the highly sensitive nature of the information collected, including information about drug use practices and locations.

## Abbreviations

HIV: Human Immunodeficiency Virus
OAT: Opioid Agonist Therapy
PWID: People who inject drugs

## References

[1] Dumchev K, Kornilova M, Kulchynska R, Azarskova M, Vitek C (2020). Improved ascertainment of modes of HIV transmission in Ukraine indicates importance of drug injecting and homosexual risk. BMC Public Health.

[2] Dumchev K, Sazonova Y, Salyuk T, Varetska O (2018). Trends in HIV prevalence among people injecting drugs, men having sex with men, and female sex workers in Ukraine. Int J STD AIDS.

[3] LaMonaca K, Dumchev K, Dvoriak S, Azbel L, Morozova O, Altice FL (2019). HIV, drug injection, and harm reduction trends in Eastern Europe and Central Asia: implications for international and domestic policy. Curr Psychiatry Rep.

[4] HIV Infection in Ukraine. Newsletter No. 50.; 2019. https://www.google.com/url?sa=t&rct=j&q=&esrc=s&source=web&cd=1&cad=rja&uact=8&ved=2ahUKEwikoPP_z4jeAhUDT98KHfuGD3MQFjAAegQICRAC&url=https%3A%2F%2Fphc.org.ua%2Fuploads%2Fdocuments%2Fc21991%2F8996daa51af04118a9a24efbe09d1c49.pdf&usg=AOvVaw3oEzBv6dZpRaT0MFy.

[5] Makarenko I, Mazhnaya A, Polonsky M (2016). Determinants of willingness to enroll in opioid agonist treatment among opioid dependent people who inject drugs in Ukraine. Drug Alcohol Depend.

[6] Barska Y, Sazonova Y (2015). Monitoring of behavior and HIV prevalence among people who inject drugs and their sexual partners.

[7] Sazonova Y, Saliuk T (2018). Main results of bio-behavioral surveillance among Key Populations.

[8] Friedman SR, Aral S (2001). Social networks, risk-potential networks, health, and disease. J Urban Health.

[9] Gyarmathy VA, Li N, Tobin KE (2011). Unprotected sex in heterosexual partnerships of injecting drug users in st. Petersburg, Russia. AIDS Behav.

[10] Uusküla A, Abel-Ollo K, Markina A, McNutt L-A, Heimer R (2012). Condom use and partnership intimacy among drug injectors and their sexual partners in Estonia. Sex Transm Infect.

[11] Heimer R, Grau LE, Curtin E, Khoshnood K, Singer M (2007). Assessment of HIV testing of urban injection drug users: implications for expansionof HIV testing and prevention efforts. Res Pract.

[12] Heimer R, White E. Estimation of the number of injection drug users in St. Petersburg, Russia. Drug Alcohol Depend. 109(1–3):79–83. 10.1016/j.drugalcdep.2009.12.010. 10.1016/j.drugalcdep.2009.12.010 PMC2875272 20060238

[13] Cepeda JA, Odinokova VA, Heimer R (2011). Drug network characteristics and HIV risk among injection drug users in Russia: the roles of trust, size, and stability. AIDS Behav.

[14] Booth RE, Lehman W, Dvoryak S, Brewster JT, Sinitsyna L (2009). Interventions with injection drug users in Ukraine. Addiction.

[15] Booth RE, Lehman W, Latkin C (2011). Individual and network interventions with injection drug users in 5 Ukraine cities. Am J Public Health.

[16] Egan JE, Frye V, Kurtz SP (2011). Migration, neighborhoods, and networks: approaches to understanding how urban environmental conditions affect Syndemic adverse health outcomes among gay, bisexual and other men who have sex with men. AIDS Behav.

[17] Tobin KE, Hester L, Davey-Rothwell MA, Latkin CA (2012). An examination of spatial concentrations of sex exchange and sex exchange norms among drug users in Baltimore, MD. Ann Assoc Am Geogr.

[18] Wylie JJL, Shah L, Jolly A (2007). Incorporating geographic settings into a social network analysis of injection drug use and bloodborne pathogen prevalence. Health Place.

[19] Tobin KEE, Cutchin M, Latkin CAA, Takahashi LMM (2013). Social geographies of African American men who have sex with men (MSM): a qualitative exploration of the social, spatial and temporal context of HIV risk in Baltimore, Maryland. Health Place.

[20] Martinez AN, Lorvick J, Kral AH (2014). Activity spaces among injection drug users in San Francisco. Int J Drug Policy.

[21] Takahashi L (1997). The socio-spatial stigmatization of homelessness and HIV/AIDS: toward an explanation of the NIMBY syndrome. Soc Sci Med.

[22] Wilton RD (1996). Diminished worlds? The geography of everyday life with HIV/AIDS. Health Place.

[23] Forsyth A, Hammersley R, Lavelle T, Murray K (1992). Geographical aspects of scoring illegal drugs. Br J Criminol.

[24] Cooper HLF, Tempalski B (2014). Integrating place into research on drug use, drug users’ health, and drug policy. Int J Drug Policy.

[25] Takahashi LM, Weibe D, Rodriquez R (2001). Navigating the time-space context of HIV and AIDS: daily routines and access to care. Soc Sci Med.

[26] Takahashi LM, Magalong MG (2008). Disruptive social capital: (un) healthy socio-spatial interactions among Filipino men living with HIV/AIDS. Health Place.

[27] Small W, Rhodes T, Wood E, Kerr T (2007). Public injection settings in Vancouver: physical environment, social context and risk. Int J Drug Policy.

[28] Sarang A, Rhodes T, Sheon N, Page K (2010). Policing drug users in Russia: risk, fear, and structural violence. Subst Use Misuse.

[29] Rhodes T, Mikhailova L, Sarang A (2003). Situational factors influencing drug injecting, risk reduction and syringe exchange in Togliatti City, Russian Federation: a qualitative study of micro risk environment. Soc Sci Med.

[30] Small W, Kerr T, Charette J, Schechter MT, Spittal PM (2006). Impacts of intensified police activity on injection drug users: evidence from an ethnographic investigation. Int J Drug Policy.

[31] Cooper H, Moore L, Gruskin S, Krieger N (2005). The impact of a police drug crackdown on drug injectors’ ability to practice harm reduction: a qualitative study. Soc Sci Med.

[32] Boodram B, Mackesy-Amiti M-E, Latkin C (2015). The role of social networks and geography on risky injection behaviors of young persons who inject drugs. Drug Alcohol Depend.

[33] Zelenev A, Long E, Bazazi AR, Kamarulzaman A, Altice FL (2016). The complex interplay of social networks, geography and HIV risk among Malaysian drug injectors: results from respondent-driven sampling. Int J Drug Policy.

[34] Rainham D, McDowell I, Krewski D, Sawada M (2010). Conceptualizing the healthscape: contributions of time geography, location technologies and spatial ecology to place and health research. Soc Sci Med.

[35] Lenhard J (2017). You care more for the gear than the geezer? Care relationships of homeless substance users in London. City Soc.

[36] Mazhnaya A, Kiriazova T, Chernova O, Tobin K, Owczarzak J (2021). “Now it is mostly done through stashes, to do it in person one has to trust you”: understanding the retail injection drug market in Dnipro, Ukraine. Int J Drug Policy.

[37] Boyd J, Richardson L, Anderson S, Kerr T, Small W, McNeil R (2018). Transitions in income generation among marginalized people who use drugs: a qualitative study on recycling and vulnerability to violence. Int J Drug Policy.

[38] DeBeck K, Shannon K, Wood E, Li K, Montaner J, Kerr T (2007). Income generating activities of people who inject drugs. Drug Alcohol Depend.

[39] Richardson L, Wood E, Kerr T (2013). The impact of social, structural and physical environmental factors on transitions into employment among people who inject drugs. Soc Sci Med.

[40] Ferguson KM, Bender K, Thompson SJ (2015). Gender, coping strategies, homelessness stressors, and income generation among homeless young adults in three cities. Soc Sci Med.

[41] Cheng T, Kerr T, Small W, Nguyen P, Wood E, DeBeck K (2016). High prevalence of risky income generation among street-involved youth in a Canadian setting. Int J Drug Policy.

[42] Owczarzak J, Kazi AK, Mazhnaya A (2021). “You’re nobody without a piece of paper:” visibility, the state, and access to services among women who use drugs in Ukraine. Soc Sci Med.

[43] Carroll J (2019). Narkomania: drugs, HIV, and citizenship in Ukraine.

[44] Bojko MJ, Mazhnaya A, Makarenko I, et al. “Bureaucracy & Beliefs”: assessing the barriers to accessing opioid substitution therapy by people who inject drugs in Ukraine. Drugs Educ Prev policy. 2015:1–8. 10.3109/09687637.2015.1016397. 10.3109/09687637.2015.1016397 PMC4831711 27087758

[45] Mazhnaya A, Bojko MJ, Marcus R, et al. In their own voices: breaking the vicious cycle of addiction, treatment and criminal justice among people who inject drugs in Ukraine. Drugs Educ Prev Policy. 2016:1–13. 10.3109/09687637.2015.1127327. 10.3109/09687637.2015.1127327 PMC4957015 27458326

[46] Richardson L, Mammel M, Milloy M-J, Hayashi K (2019). Employment cessation, Long term labour market engagement and HIV infection risk among people who inject drugs in an urban Canadian setting. AIDS Behav.

[47] Rhodes T, Kimber J, Small W (2006). Public injecting and the need for ‘safer environment interventions’ in the reduction of drug-related harm. Addiction.

[48] Tan J, Altice FL, Madden LM, Zelenev A (2020). Effect of expanding opioid agonist therapies on the HIV epidemic and mortality in Ukraine: a modelling study. Lancet HIV.

[49] Bojko MJ, Mazhnaya A, Marcus R (2016). The future of opioid agonist therapies in Ukraine: a qualitative assessment of multilevel barriers and ways forward to promote retention in treatment. J Subst Abus Treat.

[50] Kuczyńska K, Grzonkowski P, Kacprzak Ł, Zawilska JB (2018). Abuse of fentanyl: an emerging problem to face. Forensic Sci Int.

[51] Mounteney J, Giraudon I, Denissov G, Griffiths P (2015). Fentanyls: are we missing the signs? Highly potent and on the rise in Europe. Int J Drug Policy.

[52] Mazhnaya A, Tobin KE, Owczarzak J (2018). Association between injection in public places and HIV/HCV risk behavior among people who use drugs in Ukraine. Drug Alcohol Depend.

[53] Corneil T, Kuyper L, Shoveller J (2006). Unstable housing, associated risk behaviour, and increased risk for HIV infection among injection drug users. Health Place.

[54] Dickson-Gomez J, Hilario H, Convey M, Corbett AM, Weeks M, Martinez M (2009). The relationship between housing status and HIV risk among active drug users: a qualitative analysis. Subst Use Misuse.

[55] Ravn S, Duff C (2015). Putting the party down on paper: a novel method for mapping youth drug use in private settings. Health Place.

[56] Bourgois P (1998). The moral economies of homeless heroin addicts: confronting ethnography, HIV risk, and everyday violence in San Francisco shooting encampments. Subst Use Misuse.

[57] Bourgois P, Schonberg J (2009). Righteous Dopefiend.

